# Elliptic Curve Cryptography for Wireless Sensor Networks Using the Number Theoretic Transform

**DOI:** 10.3390/s20051507

**Published:** 2020-03-09

**Authors:** Utku Gulen, Selcuk Baktir

**Affiliations:** Computer Engineering Department, Faculty of Engineering and Natural Sciences, Bahcesehir University, 34353 Istanbul, Turkey; utku.gulen@eng.bau.edu.tr

**Keywords:** elliptic curve cryptography, fast Fourier transform, number theoretic transform, wireless sensor network, finite field multiplication

## Abstract

We implement elliptic curve cryptography on the MSP430 which is a commonly used microcontroller in wireless sensor network nodes. We use the number theoretic transform to perform finite field multiplication and squaring as required in elliptic curve scalar point multiplication. We take advantage of the fast Fourier transform for the first time in the literature to speed up the number theoretic transform for an efficient realization of elliptic curve cryptography. Our implementation achieves elliptic curve scalar point multiplication in only 0.65 s and 1.31 s for multiplication of fixed and random points, respectively, and has similar or better timing performance compared to previous works in the literature.

## 1. Introduction

Wireless sensor network (WSN) technology is a widespread and enabling technology that has been rapidly penetrating our daily lives. It has environmental applications such as temperature, humidity, pressure and fire monitoring [1,2], health applications such as patient monitoring [3], military applications such as enemy detection and reconnaissance [4], and applications to smart cities such as in smart grids [5]. Securing WSN applications is an important task since sensitive information they communicate should be kept confidential from malicious third parties. A sensor node, which is a single unit of a WSN, is a tiny, cheap and constrained embedded system that is usually equipped with a simple microcontroller. Cryptographic solutions are needed for applications running on constrained microcontrollers on sensor nodes [6,7,8,9,10,11,12,13]. However, due to the complex nature of cryptographic algorithms and the constrained nature of WSN nodes, e.g., their CPU power and memory size limitations, it is a challenge to implement cryptographic algorithms efficiently on WSN nodes [14,15,16,17,18].

Among different types of cryptosystems, symmetric key cryptography comes forward as a good choice to be used for WSNs due to its simplicity and efficiency. However, for many WSN applications, the distribution of the private key between sensor nodes remains as a problem that needs to be addressed. Public key cryptography (PKC) [19] provides a solution to the key distribution problem, yet it is considered computationally expensive for constrained WSN nodes. On the other hand, previous works prove PKC to be applicable on constrained WSN nodes for solving the key distribution problem [20,21,22,23,24]. Elliptic curve cryptography (ECC) [25,26] is a popular option for PKC. It requires a 160-bit or longer key to be considered secure, while the same level of security can be achieved with much longer key sizes with other PKC algorithms, e.g., a 1024-bit key is needed to achieve the same level of security using the RSA cryptosystem [27]. In this work, we realize an efficient implementation of ECC for solving the key distribution problem in WSNs. We present a novel implementation of ECC over an *optimal extension field* [28,29] by using Edwards curves [30] and the number theoretic transform [31].

The underlying finite field has a significant influence on the performance of an ECC implementation. An optimal extension field [28,29] is a finite field $GF(p^{m})$ where *p* is a pseudo-Mersenne prime of the form $p=2^{k}-c$, *k* is the processor word size and $\log_{2}\left|c\right|<\lfloor \frac{k}{2}\rfloor$. Since the coefficients of a finite field element fit in a single processor word in an optimal extension field, no multi-precision arithmetic is needed and elliptic curve point operations can be achieved efficiently. Furthermore, an irreducible field generating polynomial of the form $P\left(x\right)=x^{m}-w$, where *w* is a small integer, is used in an optimal extension field, which allows the result of a finite field multiplication operation to be reduced efficiently with only linear complexity.

The number theoretic transform (NTT), also known as the discrete Fourier transform over a finite field, has long been known for its applications in signal processing and communications [32,33,34,35,36,37]. Recently, the use of the NTT has been explored to speed up multiplication of large operands as they appear in RSA [38], fully homomorphic encryption [39,40,41,42] and post-quantum cryptography [43,44,45,46,47,48,49,50] algorithms. However, the application of the NTT for ECC has been considered impractical and widely neglected due to much shorter operands used in ECC arithmetic. There are only a few existing ECC implementations in the literature that use the NTT and they use only partial NTT computations to achieve finite field multiplication. Efficient low-area hardware implementations of ECC are given in [51,52] where finite field arithmetic is achieved in the frequency domain and partial NTT computations are performed for the modular reduction operation after a finite field multiplication. Using the same approach, in [20] an efficient implementation of ECC is presented for constrained microcontrollers. NTT-based multiplication is in general considered efficient only for large operands and believed to be not feasible for constrained microcontrollers. However, it also has the unique advantage of requiring only a linear number of word multiplications which we take advantage of in this work. Our target platform, i.e., MSP430, is a constrained microcontroller with a 16-bit RISC architecture and used widely in WSN nodes. While there is an on-board hardware multiplier on the MSP430, a word multiplication operation using the hardware multiplier still takes 14 clock cycles. Whereas, a word addition on the same microcontroller takes only a single clock cycle. Hence, by reducing the number of performed word multiplications and exchanging them with simpler operations such as addition, the speed of finite field multiplication can be significantly improved.

With this work, we use the NTT to implement finite field multiplication. We show that NTT-based finite field multiplication is feasible for small operand sizes and can be taken advantage of to speed up ECC on WSN nodes. We introduce novel implementations of the forward and inverse NTT computations over a finite field which exploit the *Fast Fourier Transform* (FFT) [53,54]. Edwards curves, introduced in [30], are a new form for elliptic curves which provide efficient formulae for elliptic curve point arithmetic. In our ECC implementation, we use the optimal extension field $GF({\left(2^{13}-1\right)}^{13})$ and Edwards curves with our improved formulae for point arithmetic that take advantage of NTT-based finite field multiplication.

**Our Main Contribution:** We present a novel realization of ECC which uses Edwards curves for point arithmetic and the NTT for the underlying finite field multiplication and squaring operations. To the best of our knowledge, our work presents the first realization of ECC using the *Fast Fourier Transform* (FFT) [53,54] to speed up NTT computations. Our implementation achieves similar or faster timings for ECC scalar point multiplication compared to existing implementations in the literature and proves that NTT-based arithmetic is feasible for ECC implementations on constrained devices such as WSN nodes.

The paper continues as follows. In Section 2, we explain ECC using Edwards curves and also give a detailed explanation of finite field multiplication in $GF({\left(2^{13}-1\right)}^{13})$ using the NTT. In Section 3, we give the details of our optimized implementation of ECC point multiplication which uses our improved Edwards curves formulae for point arithmetic and NTT-based finite field multiplication/squaring over $GF({\left(2^{13}-1\right)}^{13})$. In Section 4, we present our implementation results and comparisons with the existing work in the literature. Finally, Section 5 includes our conclusion.

## 2. Background

### 2.1. Finite Field Multiplication Using the NTT

In elliptic curve cryptography, a large number of multiplication and squaring operations are performed in a finite field. Elements of the finite field $GF(p^{m})$ are typically represented in the time domain as polynomials of degree m−1 with coefficients in GF(p) [55,56]. For instance, $a\left(x\right)\in GF\left(p^{m}\right)$ is represented as $a\left(x\right)=\sum_{i=0}^{m-1}a_{i}x^{i}=a_{0}+a_{1}x+a_{2}x^{2}+\ldots +a_{m-1}x^{m-1}$, where $a_{i}\in GF\left(p\right)$ for 0≤i≤m−1. Multiplication of two $GF(p^{m})$ elements, e.g., a(x) and b(x), is achieved typically by computing the polynomial product

$$c^{\prime }\left(x\right)=a\left(x\right)\cdot b\left(x\right)\;\mathrm{mod}\;p$$

followed by the modular reduction

$$c\left(x\right)=c^{\prime }\left(x\right)\;\mathrm{mod}\;P(x)\;,$$

where P(x) is the irreducible field generating polynomial. Please note that if the field generating polynomial can be selected as the binomial $x^{m}-2$, the cost of the modular reduction computation becomes negligible. Due to the convolution theorem, the classical polynomial multiplication operation in the time domain, e.g., the computation of $c^{\prime }\left(x\right)=a\left(x\right)\cdot b\left(x\right)\;\mathrm{mod}\;p$, which has quadratic complexity, is equivalent to the simple pairwise multiplication of the corresponding frequency domain sequence coefficients which has only linear complexity [53]. Thus, the complexity of polynomial multiplication can be reduced by performing this computation in the frequency domain.

The coefficients of a $GF(p^{m})$ element form a time domain sequence. To perform the polynomial multiplication of two $GF(p^{m})$ elements in the frequency domain, the time domain sequences for the two $GF(p^{m})$ elements should be transformed into their corresponding frequency domain sequences. This conversion is achieved by using the NTT [31]. After the polynomial multiplication operation is completed in the frequency domain, the result can be converted back to the time domain by using the inverse NTT computation. Algorithm 1 gives an overview of how polynomial multiplication can be achieved in the frequency domain.
**Algorithm 1:** Polynomial Multiplication in the Frequency Domain Using the NTT **Input**: (a) and (b), the time domain sequences for $a\left(x\right),b\left(x\right)\in GF\left(p^{m}\right)$ **Output**: $\left(c^{\prime }\right)$, the time domain sequence for $c^{\prime }\left(x\right)=a\left(x\right)\cdot b\left(x\right)\;\mathrm{mod}\;p$**_1_**(A)⟵NTT((a))     //Compute the NTT of (a)**_2_**(B)⟵NTT((b))     //Compute the NTT of (b)**_3_**$\;\left(C^{\prime }\right)⟵PCM\left(\left(A\right),\left(B\right)\right)$  //Pairwise Coefficient Multiplication**_4_**$\;\;\left(c^{\prime }\right)⟵INTT\left(\left(C^{\prime }\right)\right)$    //Compute the inverse NTT of (C’)**_5_ Return**$\left(c^{\prime }\right)$

A finite field element $a\left(x\right)\in GF\left(p^{m}\right)$ can be converted to its *d*-element frequency domain sequence representation, where d≥m, in two steps as explained below:

1. Represent $a\left(x\right)=a_{0}+a_{1}x+a_{2}x^{2}+\ldots +a_{m-1}x^{m-1}$ as the following time domain sequence
  (1) $$\left(a\right)=\left(a_{0},a_{1},a_{2},\ldots ,a_{m-1},0,0,\ldots ,0\right)$$
  by appending d−m zeros at the end.
2. Obtain the frequency domain sequence representation $\left(A\right)=\left(A_{0},A_{1},A_{2},\ldots ,A_{d-1}\right)$ for a(x) by performing the following NTT computation over (a):
  (2) $$A_{j}=\sum_{i=0}^{d-1}a_{i}r^{ij}\;\mathrm{mod}\;p,\;0\leq j\leq d-1\;,$$
  where *r* is a $d^{th}$ primitive root of unity.

Please note that in order to obtain the time domain sequence (a) back from the frequency domain sequence (A), the *inverse* NTT computation can be used as follows:

(3) $$a_{i}=\frac{1}{d}\cdot \sum_{j=0}^{d-1}A_{j}r^{-ij}\;\mathrm{mod}\;p\;\;,\;0\leq i\leq d-1\;.$$

In Algorithm 1, since $c^{\prime }\left(x\right)=a\left(x\right)\cdot b\left(x\right)\;\mathrm{mod}\;p$ may have up to 2m−1 coefficients, representing it in the frequency domain with a sequence of length shorter than 2m−1 may result in its value being corrupted. Therefore, for Algorithm 1 to always generate the correct result, one should have d≥2m−1 as the NTT length. We now describe with an example the execution of Algorithm 1 for computing $c^{\prime }\left(x\right)=a\left(x\right)\cdot b\left(x\right)\;\mathrm{mod}\;p$ where $a\left(x\right),b\left(x\right)\in GF\left(p^{13}\right)$. As described in (1), the polynomials $a\left(x\right)=a_{0}+a_{1}x+a_{2}x^{2}+\ldots +a_{12}x^{12}$ and $b\left(x\right)=b_{0}+b_{1}x+b_{2}x^{2}+\ldots +b_{12}x^{12}$ are first converted into their corresponding 26-element time domain sequence representations as

$$\left(a\right)=\left(a_{0},a_{1},a_{2},a_{3},a_{4},a_{5},a_{6},a_{7},a_{8},a_{9},a_{10},a_{11},a_{12},0,0,0,0,0,0,0,0,0,0,0,0,0\right)$$

and

$$\left(b\right)=\left(b_{0},b_{1},b_{2},b_{3},b_{4},b_{5},b_{6},b_{7},b_{8},b_{9},b_{10},b_{11},b_{12},0,0,0,0,0,0,0,0,0,0,0,0,0\right)\;.$$

Secondly, the NTT is applied to (a) and (b), as described in (2), to obtain the following frequency domain sequences:

$$\left(A\right)=\left(A_{0},A_{1},A_{2},\cdots ,A_{23},A_{24},A_{25}\right)\;,$$

$$\left(B\right)=\left(B_{0},B_{1},B_{2},\cdots ,B_{23},B_{24},B_{25}\right)\;.$$

Thirdly, the coefficients of (A) and (B) are pairwise multiplied in the frequency domain, i.e., by computing $C_{i}^{\prime }=A_{i}B_{i}\;\mathrm{mod}\;p$ for 0≤i≤25, and thus the following sequence is obtained in the frequency domain:

$$\left(C^{\prime }\right)=\left(C_{0}^{\prime },C_{1}^{\prime },C_{2}^{\prime },C_{3}^{\prime },C_{4}^{\prime },C_{5}^{\prime },\cdots ,C_{21}^{\prime },C_{22}^{\prime },C_{23}^{\prime },C_{24}^{\prime },C_{25}^{\prime }\right)\;,$$

which corresponds to $c^{\prime }\left(x\right)=a\left(x\right)\cdot b\left(x\right)\;\mathrm{mod}\;p$ in the time domain. Finally, the inverse NTT is applied to $\left(C^{\prime }\right)$, as described in (3), to obtain the following time domain sequence for $c^{\prime }\left(x\right)=a\left(x\right)\cdot b\left(x\right)\;\mathrm{mod}\;p$:

(4) $$\left(c^{\prime }\right)=\left(c_{0}^{\prime },c_{1}^{\prime },c_{2}^{\prime },c_{3}^{\prime },c_{4}^{\prime },c_{5}^{\prime },\cdots ,c_{21}^{\prime },c_{22}^{\prime },c_{23}^{\prime },c_{24}^{\prime },0\right)\;.$$

Please note that since $c^{\prime }\left(x\right)$ is a polynomial of degree 24, $c_{25}^{\prime }$ is zero and the first 25 coefficients of $\left(c^{\prime }\right)$ give us the polynomial $c^{\prime }\left(x\right)=a\left(x\right)\cdot b\left(x\right)\;\mathrm{mod}\;p$, given as follows:

(5) $$c^{\prime }\left(x\right)=c_{0}^{\prime }+c_{1}^{\prime }x+c_{2}^{\prime }x^{2}+\cdots +c_{22}^{\prime }x^{22}+c_{23}^{\prime }x^{23}+c_{24}^{\prime }x^{24}\;.$$

As a final step in $GF(p^{13})$ multiplication, the polynomial $c^{\prime }\left(x\right)$ needs to be reduced modulo the field generating polynomial by computing $c\left(x\right)=c^{\prime }\left(x\right)\;\mathrm{mod}\;P\left(x\right)$, which has only linear complexity.

### 2.2. Elliptic Curve Cryptography Using Edwards Curves

The main operation in ECC is scalar point multiplication, i.e., computing s·P for an integer *s* and a point *P* on the elliptic curve. ECC scalar point multiplication involves performing several ECC point addition and doubling operations. To achieve ECC point multiplication, the binary method [57] can be used, where the bits of the scalar *s* are scanned one bit at a time starting with the most significant bit, and for each scanned bit, a point doubling operation is performed, in addition to a point addition operation if the scanned bit is 1. However, the binary method is both inefficient and vulnerable against simple power analysis [58]. As an alternative to the binary method, and in order to help mitigate its drawbacks, the NAF4 and Comb methods can be used for ECC scalar point multiplication of random and fixed points, respectively. NAF4 and Comb require computing a significantly reduced number point additions and doublings compared to the binary method [59].

Edwards curves, proposed in [30], are a new form for elliptic curves and defined by the following equation:

$$x^{2}+y^{2}=c^{2}\left(1+dx^{2}y^{2}\right)\;.$$

The ECC point addition of the two distinct points $P_{1}$ and $P_{2}$ on an Edwards curve is computed as

$$P_{3}\left(x_{3},y_{3}\right)=P_{1}\left(x_{1},y_{1}\right)+P_{2}\left(x_{2},y_{2}\right)\;,$$

$$\mathrm{where}\;\;\;\;x_{3}=\frac{x_{1}y_{2}+y_{1}x_{2}}{c(1+dx_{1}x_{2}y_{1}y_{2})}\;\;\;\;\mathrm{and}\;\;\;\;y_{3}=\frac{y_{1}y_{2}-x_{1}x_{2}}{c(1-dx_{1}x_{2}y_{1}y_{2})}\;.$$

The ECC point doubling operation on the point $P_{1}\left(x_{1},y_{1}\right)$ on an Edwards curve is computed as

$$P_{2}\left(x_{2},y_{2}\right)=2\cdot P_{1}\left(x_{1},y_{1}\right)\;,$$

$$\mathrm{where}\;\;\;\;x_{2}=\frac{2x_{1}y_{1}c}{x_{1}^{2}+y_{1}^{2}}\;\;\;\;\mathrm{and}\;\;\;\;y_{2}=\frac{(y_{1}^{2}-x_{1}^{2})c}{2c^{2}-\left(x_{1}^{2}+y_{1}^{2}\right)}\;.$$

The above ECC point operations can be achieved in *projective coordinates* [59,60,61] to avoid costly inversions. For the Edwards curve $x^{2}+y^{2}=c^{2}\left(1+dx^{2}y^{2}\right)$, with c=1, *d* random and $d\cdot c^{4}\neq 1$, the formulae for ECC point doubling and addition in projective coordinates over prime fields are given in Algorithms 2 and 3, respectively [62].
**Algorithm 2:** Elliptic curve point doubling in projective coordinates over prime fields using Edwards curves [62] **Input**: $P_{1}\left(X_{1}:Y_{1}:Z_{1}\right)$ **Output**: $P_{2}\left(X_{2}:Y_{2}:Z_{2}\right)=2\cdot P_{1}$**_1_** $T_{1}\leftarrow X_{1}$, $T_{2}\leftarrow Y_{1}$, $T_{3}\leftarrow Z_{1}$       **_9_** $T_{2}⟵T_{1}-T_{2}$**_2_** $T_{4}⟵T_{1}+T_{2}$              **_10_** $T_{4}⟵T_{4}-T_{5}$**_3_** $T_{1}⟵T_{1}^{2}$                **_11_** $T_{3}⟵T_{5}-T_{3}$**_4_** $T_{2}⟵T_{2}^{2}$                **_12_** $T_{1}⟵T_{3}\cdot T_{4}$**_5_** $T_{3}⟵T_{3}^{2}$                **_13_** $T_{3}⟵T_{3}\cdot T_{5}$**_6_** $T_{4}⟵T_{4}^{2}$                **_14_** $T_{2}⟵T_{2}\cdot T_{5}$**_7_** $T_{3}⟵2\cdot T_{3}$               **_15_** $T_{2}\leftarrow T_{1},Y_{2}\leftarrow T_{2},Z_{2}\leftarrow T_{3}$**_8_** $T_{5}⟵T_{1}+T_{2}$              **_16_ Return**$\left(X_{2}:Y_{2}:Z_{2}\right)$

**Algorithm 3:** Elliptic curve point addition in projective coordinates over prime fields using Edwards curves [62] **Input**: $P_{1}\left(X_{1}:Y_{1}:Z_{1}\right)$ and $P_{2}\left(X_{2}:Y_{2}:Z_{2}\right)$ **Output**: $P_{3}\left(X_{3}:Y_{3}:Z_{3}\right)=P_{1}+P_{2}$**_1_** $T_{1}\leftarrow X_{1}$, $T_{2}\leftarrow Y_{1}$, $T_{3}\leftarrow Z_{1}$, $T_{4}\leftarrow X_{2}$, $T_{5}\leftarrow Y_{2}$,   **_12_** $T_{8}⟵d\cdot T_{8}$  $T_{6}\leftarrow Z_{2}$                     **_13_** $T_{2}⟵T_{2}-T_{1}$**_2_** $T_{3}⟵T_{3}\cdot T_{6}$                   **_14_** $T_{2}⟵T_{2}\cdot T_{3}$**_3_** $T_{7}⟵T_{1}+T_{2}$                  **_15_** $T_{3}⟵{\left(T_{3}\right)}^{2}$**_4_** $T_{8}⟵T_{4}+T_{5}$                  **_16_** $T_{1}⟵T_{3}-T_{8}$**_5_** $T_{1}⟵T_{1}\cdot T_{4}$                   **_17_** $T_{3}⟵T_{3}+T_{8}$**_6_** $T_{2}⟵T_{2}\cdot T_{5}$                   **_18_** $T_{2}⟵T_{2}\cdot T_{3}$**_7_** $T_{7}⟵T_{7}\cdot T_{8}$                   **_19_** $T_{3}⟵T_{3}\cdot T_{1}$**_8_** $T_{7}⟵T_{7}-T_{1}$                  **_20_** $T_{1}⟵T_{1}\cdot T_{7}$**_9_** $T_{7}⟵T_{7}-T_{2}$                  **_21_** $X_{3}\leftarrow T_{1}$, $Y_{3}\leftarrow T_{2}$, $Z_{3}\leftarrow T_{3}$**_10_** $T_{7}⟵T_{7}\cdot T_{3}$                  **_22_** **Return**$\left(X_{3}:Y_{3}:Z_{3}\right)$**_11_** $T_{8}⟵T_{1}\cdot T_{2}$

## 3. Our ECC Implementation Using the NTT and Edwards Curves

We implement ECC over an *optimal extension field* [29,63], namely $GF(p^{m})$ with the Mersenne prime field characteristic $p=2^{13}-1$ and the prime field extension degree m=13. Please note that ECC over a prime extension field of the form $GF(p^{m})$ is considered secure when the finite field is sufficiently large and its extension degree *m* is a prime number [59]. We select the field characteristic *p* such that polynomial coefficients fit in a single processor word, in our case a 16-bit word, eliminating the need for performing multiprecision arithmetic. We use the binomial $x^{13}-2$ as the field generating polynomial which facilitates efficient modular reduction. For finite field multiplication and squaring, we use the NTT and use the approach described in Algorithm 1. For NTT computations, we use the NTT length of d=26 and the 26^th^ primitive root of unity as r=−2. We use the FFT [53,54,64] to speed up NTT computations. For ECC point doubling and addition, we use our improved versions of Algorithms 2 and 3, respectively. Finally, we use the NAF and Comb methods, with a 4-bit window, to perform ECC scalar point multiplication with random and fixed points, respectively [59].

### 3.1. Finite Field Multiplication and Squaring in $GF({\left(2^{13}-1\right)}^{13})$ with the NTT

As explained in Algorithm 1, polynomial multiplication, which is the main operation in $GF({\left(2^{13}-1\right)}^{13})$ multiplication, can be achieved using the NTT in three stages: (1) Forward NTT Computation, (2) Pairwise Coefficient Multiplication, (3) Inverse NTT Computation. We apply the FFT [53,54,64] to speed up our NTT and inverse NTT computations.

*Forward NTT for Converting $GF({\left(2^{13}-1\right)}^{13})$ Elements to the Frequency Domain:*

As described in (2), the frequency domain sequence representation $\left(A\right)=\left(A_{0},A_{1},A_{2},\ldots ,A_{25}\right)$ of $a\left(x\right)\in GF({\left(2^{13}-1\right)}^{13})$ is obtained by computing the NTT of the corresponding 26-element time domain sequence $\left(a\right)=\left(a_{0},a_{1},a_{2},\ldots ,a_{12},0,0,\cdots ,0\right)$ as

(6) $$A_{j}=\sum_{i=0}^{25}a_{i}r^{ij}\;\mathrm{mod}\;p,\;0\leq j\leq 25\;,$$

where $p=2^{13}-1$. The above NTT computation can be optimized by applying the FFT as

(7) $$A_{j}=\sum_{i=0}^{12}a_{2i}{\left(r^{2}\right)}^{ij}+r^{j}\sum_{i=0}^{12}a_{2i+1}{\left(r^{2}\right)}^{ij}\;\;\;\mathrm{mod}\;p$$

and

(8) $$A_{j+13}=\sum_{i=0}^{12}a_{2i}{\left(r^{2}\right)}^{ij}-r^{j}\sum_{i=0}^{12}a_{2i+1}{\left(r^{2}\right)}^{ij}\;\;\;\mathrm{mod}\;p\;,$$

for 0≤j≤12 [64]. Please note that the first summations in (7) and (8) are the same NTT computation. Likewise, the second summations in (7) and (8) are also the same NTT computation. Both NTT computations are of length 13. Hence, using the FFT, the computation in (6), which is a 26-element NTT computation, is reduced to the computation of roughly two 13-element NTT computations. Since $a_{i}=0$ for 13≤i≤25, we compute the summations in (7) and (8) only for *i* running from 0 to 6 in the first NTT computation, and from 0 to 5 in the second. Our optimized algorithm for computing the forward NTT of $a\left(x\right)\in GF({\left(2^{13}-1\right)}^{13})$ on the MSP430 is given in Algorithm 4.
**Algorithm 4:** Forward NTT Computation on the MSP430 Using the FFT
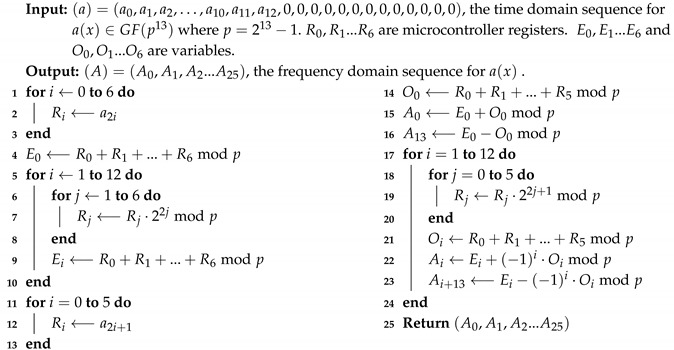


We implement Algorithm 4 with an assembly routine and optimize it by using microcontroller registers as much as possible to minimize the number of memory read/write operations. For the additions in lines 4,9,14 and 21, there is no need to do modular reduction after every addition. We reduce the number of modular reductions by accumulating the sums and deferring modular reduction as much as possible.

Multiplication of a $GF(2^{13}-1)$ element with a power of 2, e.g., in lines 7 and 19 of Algorithm 4, corresponds to a bitwise left rotation of the $GF(2^{13}-1)$ element. For instance, for $R\in GF(2^{13}-1)$, $2^{j}R\;\mathrm{mod}\;2^{13}-1$ can be computed by rotating the bits of *R* by jmod13 bits to the left. We realize multiplications of $GF(2^{13}-1)$ elements with powers of 2 with an optimized assembly routine.

Please note that for multiplying two distinct $GF({\left(2^{13}-1\right)}^{13})$ elements with Algorithm 1, Algorithm 4 needs to be executed twice, i.e., once for each input operand to obtain its frequency domain sequence representation. On the other hand, for squaring a $GF({\left(2^{13}-1\right)}^{13})$ element, Algorithm 4 needs to be executed only once for the single input operand. Hence, squaring using Algorithm 1 is faster than multiplication.

*Pairwise Coefficient Multiplication of $GF({\left(2^{13}-1\right)}^{13})$ Elements in the Frequency Domain:*

Polynomial multiplication of two $GF({\left(2^{13}-1\right)}^{13})$ elements can be achieved in the frequency domain with only linear complexity by multiplying pairwise their frequency domain sequence coefficients. Let $a\left(x\right),b\left(x\right)\in GF({\left(2^{13}-1\right)}^{13})$, and let $\left(A\right)=\left(A_{0},A_{1},\cdots ,A_{25}\right)$ and $\left(B\right)=\left(B_{0},B_{1},\cdots ,B_{25}\right)$ be their 26-element frequency domain sequence representations obtained using Algorithm 4. The following 26 pairwise coefficient multiplications generate the 26-element frequency domain sequence representation (C’) of the product $c^{\prime }\left(x\right)=a\left(x\right)\cdot b\left(x\right)\;\mathrm{mod}\;p$:

(9) $$C_{i}^{\prime }=A_{i}B_{i}\;\;\;\mathrm{mod}\;p\;,\;\;\;\;0\leq i\leq 25\;.$$

The frequency domain sequence $\left(C^{\prime }\right)$ can be converted back to the time domain, by applying the inverse NTT, to give us the coefficients of the polynomial product $c^{\prime }\left(x\right)=a\left(x\right)\cdot b\left(x\right)\;\mathrm{mod}\;p$.

The multiplications in (9) are the only GF(p) multiplications required for computing the polynomial product $c^{\prime }\left(x\right)=a\left(x\right)\cdot b\left(x\right)\;\mathrm{mod}\;p$ in the NTT-based multiplication approach. Please note that only 26 coefficient multiplications are performed here, which is significantly less than the 169 coefficient multiplications required in the classical schoolbook method for multiplication.

*Inverse NTT for Converting the Frequency Domain Product to a Time Domain $GF({\left(2^{13}-1\right)}^{13})$ Element:*

As described in (3), the time domain sequence representation $\left(c^{\prime }\right)=\left(c_{0}^{\prime },c_{1}^{\prime },c_{2}^{\prime },\ldots ,c_{25}^{\prime }\right)$ of $c^{\prime }\left(x\right)=a\left(x\right)\cdot b\left(x\right)\;\mathrm{mod}\;p$ can be obtained by computing the inverse NTT of the corresponding 26-element frequency domain sequence $\left(C^{\prime }\right)=\left(C_{0}^{\prime },C_{1}^{\prime },C_{2}^{\prime },\cdots ,C_{23}^{\prime },C_{24}^{\prime },C_{25}^{\prime }\right)$ as follows

(10) $$c_{j}^{\prime }=\frac{1}{26}\sum_{i=0}^{25}C_{i}^{\prime }r^{-ij}\;\;\mathrm{mod}\;p\;,\;\;\;\;0\leq j\leq 25\;.$$

The above inverse NTT computation can be optimized by applying the inverse FFT as

(11) $$c_{j}^{\prime }=\sum_{i=0}^{12}C_{2i}^{\prime }{\left(r^{2}\right)}^{-ij}+r^{-j}\sum_{i=0}^{12}C_{2i+1}^{\prime }{\left(r^{2}\right)}^{-ij}\;\;\;\mathrm{mod}\;p$$

and

(12) $$c_{j+13}^{\prime }=\sum_{i=0}^{12}C_{2i}^{\prime }{\left(r^{2}\right)}^{-ij}-r^{-j}\sum_{i=0}^{12}C_{2i+1}^{\prime }{\left(r^{2}\right)}^{-ij}\;\;\;\mathrm{mod}\;p\;,$$

for 0≤j≤12 [64]. Please note that the first summations in (11) and (12) are the same inverse NTT computation. Likewise, the second summations in (11) and (12) are the same inverse NTT computation. Furthermore, both inverse NTT computations are of length 13. Hence, using the inverse FFT, the computation of (10), which is a 26-element inverse NTT computation, is reduced to the computation of roughly two 13-element inverse NTT computations. Since $c_{25}^{\prime }=0$, we compute the second summations in (11) and (12) only for *i* running from 0 to 11. Our inverse FFT algorithm for computing the inverse NTT of $\left(C^{\prime }\right)$, the frequency domain sequence corresponding to $c^{\prime }\left(x\right)=a\left(x\right)\cdot b\left(x\right)\;\mathrm{mod}\;p$, and for obtaining $c\left(x\right)=c^{\prime }\left(x\right)\;\mathrm{mod}\;P\left(x\right)$, where $P\left(x\right)=x^{13}-2$, is given in Algorithm 5.

Please note that, unlike in the inverse NTT computation in Algorithm 1, in Algorithm 5 (lines 35−42) we embed the modular reduction of $c^{\prime }\left(x\right)=a\left(x\right)\cdot b\left(x\right)\;\mathrm{mod}\;p$ by the field generating polynomial $P\left(x\right)=x^{13}-2$. Hence, for $a\left(x\right),b\left(x\right)\in GF({\left(2^{13}-1\right)}^{13})$, while the output of Algorithm 1 is a polynomial of degree 24 (with 25 coefficients in $GF(2^{13}-1)$, the output of Algorithm 5 is an element of $GF({\left(2^{13}-1\right)}^{13})$ with 13 coefficients.

Similar to our implementation of Algorithm 4, we implement Algorithm 5 with an assembly routine and optimize it by using microcontroller registers exhaustively to minimize the number of memory read/write operations. We reduce the number of performed modular reductions in lines 4,9,14,19,24,29,34 and 40 of Algorithm 5 by accumulating the sums and deferring the modular reduction computation as much as possible.

Division of a $GF(2^{13}-1)$ element by a power of 2, e.g., in lines 7,17,27 and 38 of Algorithm 5, can be achieved with a bitwise right rotation. For instance, for $R\in GF(2^{13}-1)$, $R/2^{j}\;\mathrm{mod}\;2^{13}-1$ can be computed by rotating the bits of *R* by jmod13 bits to the right. We realize this bitwise rotation operation with an optimized assembly routine.
**Algorithm 5:** Inverse NTT Computation on the MSP430 Using the FFT
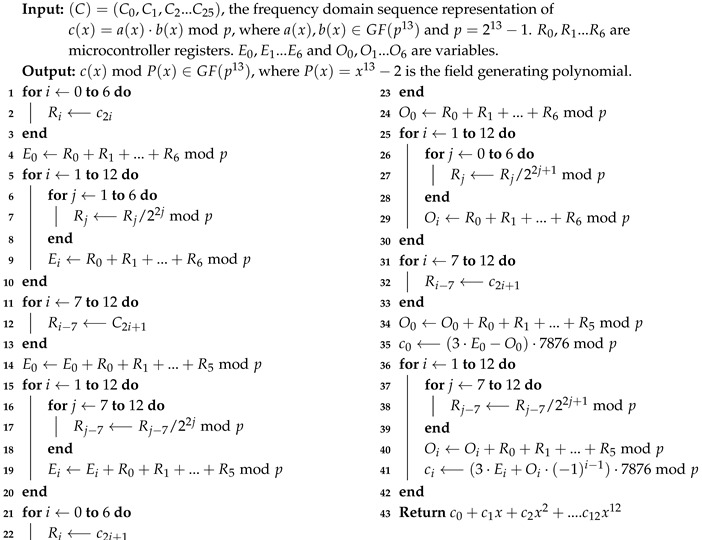


### 3.2. ECC Point Arithmetic with NTT Based Multiplication and Squaring

For ECC operations, we use the Edwards curve $x^{2}+y^{2}=c^{2}\left(1+dx^{2}y^{2}\right)$, with c=1, *d* random and $d\cdot c^{4}\neq 1$, over the 169-bit prime extension field $GF({\left(2^{13}-1\right)}^{13})$, and use our optimized versions of the elliptic curve point addition and doubling formulae given in Algorithms 2 and 3. We improve Algorithms 2 and 3 by taking advantage of NTT-based multiplication and squaring operations. Our improved algorithms are given in Algorithms 6 and 7.
**Algorithm 6:** Elliptic curve point doubling in projective coordinates over prime fields using Edwards curves and NTT-based multiplication/squaring **Input**: $P=(X_{1}:Y_{1}:Z_{1})$, $R_{1}$ and $R_{2}$ are temporary registers. **Output**: $2P=(X_{2}:Y_{2}:Z_{2})$**_1_** $R_{1}⟵NTT\left(X_{1}\right)+NTT\left(Y_{1}\right)$//NTTsstored  **_8_** $R_{1}⟵R_{1}-R_{2}$**_2_** $R_{1}⟵{R_{1}}^{2}$                      **_9_** $Z_{1}⟵R_{2}-Z_{1}$**_3_** $X_{1}⟵{X_{1}}^{2}$                      **_10_** $X_{2}⟵Z_{1}\cdot R_{1}$//NTTof$Z_{1}$stored**_4_** $Y_{1}⟵{Y_{1}}^{2}$                      **_11_** $Z_{2}⟵Z_{1}\cdot R_{2}$//NTTof$R_{2}$stored**_5_** $Z_{1}⟵2{Z_{1}}^{2}$                     **_12_** $Y_{2}⟵Y_{1}\cdot R_{2}$**_6_** $R_{2}⟵X_{1}+Y_{1}$                   **_13_** **Return**$\left(X_{2}:Y_{2}:Z_{2}\right)$**_7_** $Y_{1}⟵X_{1}-Y_{1}$

Algorithm 6 is a reordered and optimized version of Algorithm 2. It takes advantage of NTT-based finite field multiplication and squaring computations. In line 1 of the algorithm, the NTTs of $X_{1}$ and $Y_{1}$ are computed, and then added in the frequency domain to find the NTT of $R_{1}=X_{1}+Y_{1}$. The computed NTTs of $X_{1},Y_{1}$ and $R_{1}$ are stored. The stored frequency domain representations of $X_{1},Y_{1}$ and $R_{1}$ are used in lines 2−4 (marked bold) for the three finite field squarings. Please note that for these three finite field squarings, a total number of only two forward NTT computations are performed, i.e., $NTT(X_{1})$ and $NTT(Y_{1})$ in line 1, instead of three as required in Algorithm 1. Furthermore, in line 10, the computed NTT of $Z_{1}$ is stored and reused in line 11 (marked bold). Similarly, in line 11, the computed NTT of $R_{2}$ is stored and reused in line 12 (marked bold). Please note that each time the stored result of an NTT computation is reused, a forward NTT computation is saved in Algorithm 1.
**Algorithm 7:** Elliptic curve point addition in projective coordinates over prime fields using Edwards curves and NTT-based multiplication/squaring **Input**: $P=(X_{1}:Y_{1}:Z_{1})$, $Q=(X_{2}:Y_{2}:Z_{2})$, $R_{1}$ and $R_{2}$ are temporary registers. **Output**: $P+Q=(X_{3}:Y_{3}:Z_{3})$**_1_** 
$Z_{1}⟵Z_{1}\cdot Z_{2}$                  **_11_** $Y_{1}⟵Y_{1}-X_{1}$
//NTT
of
$Y_{1}$
stored**_2_** $R_{1}⟵NTT\left(X_{1}\right)+NTT\left(Y_{1}\right)$//NTTsstored  **_12_** $Y_{1}⟵Y_{1}\cdot Z_{1}$**_3_** $R_{2}⟵NTT\left(X_{2}\right)+NTT\left(Y_{2}\right)$//NTTsstored  **_13_** $Z_{1}⟵{Z_{1}}^{2}$**_4_** $R_{1}⟵R_{1}\cdot R_{2}$                  **_14_** $X_{1}⟵Z_{1}-R_{2}$**_5_** $X_{1}⟵X_{1}\cdot X_{2}$                  **_15_** $Z_{1}⟵Z_{1}+R_{2}$**_6_** $Y_{1}⟵Y_{1}\cdot Y_{2}$                  **_16_** $Y_{3}⟵Y_{1}\cdot Z_{1}$//NTTof$Z_{1}$stored**_7_** $R_{1}⟵R_{1}-X_{1}$                 **_17_** $Z_{3}⟵Z_{1}\cdot X_{1}$//NTTof$X_{1}$stored**_8_** $R_{1}⟵R_{1}-Y_{1}$                  **_18_** $X_{3}⟵X_{1}\cdot R_{1}$**_9_** $R_{1}⟵R_{1}\cdot Z_{1}$//NTTof$Z_{1}$stored     **_19_** **Return**$\left(X_{3}:Y_{3}:Z_{3}\right)$**_10_** $R_{2}⟵d\cdot X_{1}\cdot Y_{1}$//NTTsof$X_{1}$and$Y_{1}$stored

Algorithm 7 is a reordered and optimized version of Algorithm 3. It takes advantage of NTT-based finite field multiplication and squaring computations. In lines 2−3 of the algorithm, the NTTs of $X_{1},X_{2},Y_{1}$ and $Y_{2}$ are computed and stored. Only two addition operations are performed in the frequency domain on the stored NTTs to readily obtain the NTTs of $R_{1}=X_{1}+Y_{1}$ and $R_{2}=X_{2}+Y_{2}$. The NTTs of $R_{1}$ and $R_{2}$ are also stored. The stored NTTs of $R_{1},R_{2},X_{1},X_{2},Y_{1}$ and $Y_{2}$ are readily used in lines 4−6 (denoted with bold color) for the three finite field multiplication computations. Thus, for three finite field multiplications, a total number of only four forward NTT computations are performed, instead of six as required in Algorithm 1. Furthermore, in lines 11−13 of the algorithm, the stored NTTs of $Y_{1},X_{1}$ and $Z_{1}$ are reused (marked bold). Similarly, in line 16, the NTT of $Z_{1}$ is computed and stored. The stored NTT of $Z_{1}$ is reused in line 17 (marked bold). Likewise, in line 17, the NTT of $X_{1}$ is computed and stored, and reused in line 18 (marked bold).

## 4. Implementation Results

We use Texas instrument’s MSP430 microcontroller, which is commonly used in wireless sensor nodes, and select version MSP430F1611 [65]. Our target device, MSP430F1611, is a 1-series low power microcontroller which runs at 8 MHz clock frequency, and has a 48 kB flash memory in addition to a 10 kB RAM. We develop our code in the C language but also use the assembly language for computationally intensive and/or commonly performed operations. We use the IAR Workbench IDE as our development environment [66]. We obtain timings by using the IAR Workbench IDE’s clock cycle counter in debug mode. The detailed timing figures for our ECC implementations are given in Table 1.

**Figure 1:**
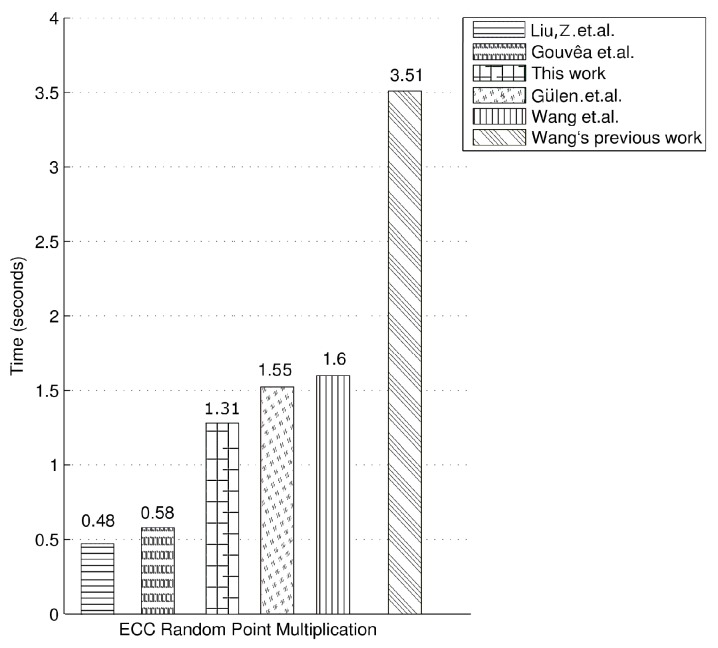
Comparison for random point multiplication timings.

In Table 2 and Figure 1, we present our timings for ECC random point multiplication on the MSP430F1611 as well as the timings of the related work in the literature on the same microcontroller. Liu et al.’s work, which uses a 159-bit Montgomery curve, presents the fastest timing for random point multiplication on the MSP430 microcontroller [67]. They use the Montgomery ladder method and achieve random point multiplication in 3,460,000 clock cycles which is equivalent to 0.48 s at 8 MHz clock frequency. Gouvêa et al.’s work, which uses the 160-bit curve secp160r1 that has a slightly smaller elliptic curve group order than ours, achieves ECC random point multiplication in 0.58 s [68]. Our previous ECC implementation over $GF({\left(2^{13}-1\right)}^{13})$ on the MSP430F149, a similar microcontroller to the MSP430F1611, achieves random point multiplication in 1.55 s [20]. Please note that our ECC random point multiplication implementation in this work, which exploits the NTT-based finite field multiplication/squaring and the FFT, is more than 18% faster than our previous implementation on the same elliptic curve. Wang et al.’s implementation of elliptic curve random point multiplication over a 160-bit elliptic curve has a timing value of 3.51 s which is significantly worse than our timing result [69]. In a later work, the same authors improve their timing to 1.60 s; however, their new implementation is still 22% slower than our work [24]. Please note that the timing figure for our ECC implementation is for a 169-bit elliptic curve with a higher security level, whereas the others’ works use the smaller ordered 159-bit and 160-bit elliptic curves.

**Figure 2:**
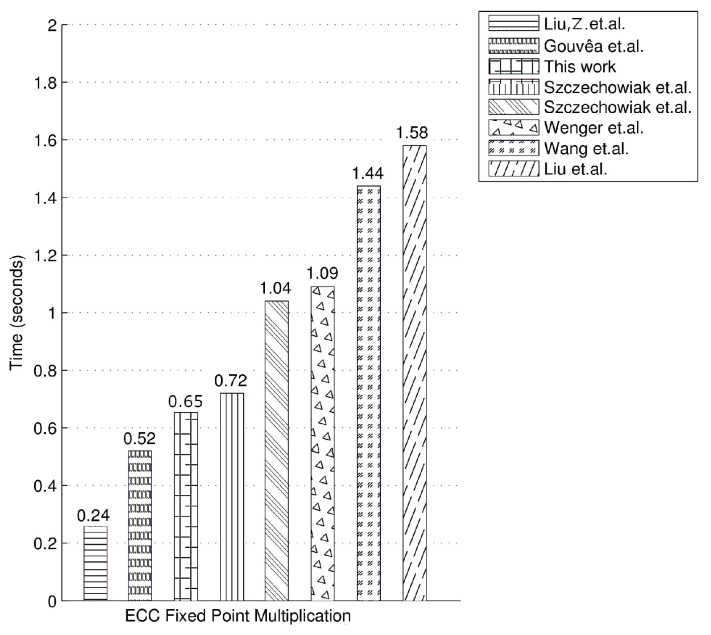
Comparison for fixed point multiplication timings.

In Table 3 and Figure 2, we present our timings for ECC fixed point multiplication on the MSP430F1611 as well as the timings of the related work in the literature on the same microcontroller. Liu et al.’s work, which uses a 159-bit twisted Edwards curve, presents the fastest timing for fixed point multiplication on the MSP430 microcontroller [67]. They use the Comb method and twisted Edwards curves to achieve fixed point multiplication in 1,920,000 clock cycles which is equivalent to 0.24 s at 8 MHz clock frequency. Gouvêa et al.’s work, which uses the 160-bit elliptic curve secp160r1 and the 4NAF method, achieves ECC fixed point multiplication in 0.52 s [68]. Liu et al.’s timing for 160-bit ECDSA signature generation (considered to have around the same timing value as elliptic curve fixed point multiplication) is 1.58 s, which is twice slower than our implementation that uses a larger 169-bit elliptic curve. Wang et al.’s work on the same microcontroller achieves elliptic curve fixed point multiplication in 1.44 s over a 160-bit elliptic curve. Wenger et al.’s implementation of elliptic curve fixed point multiplication on a 160-bit elliptic curve takes 8,779,931 clock cycles which is equivalent to 1.09 s at 8 MHz clock frequency [70]. Szczechowiak et al.’s work achieves elliptic curve fixed point multiplication in 0.72 s using a 160-bit elliptic curve over a prime field [22] and in 1.04 s using a 163-bit elliptic curve over a binary field [22]. Our timing for elliptic curve fixed point multiplication over a larger ordered 169-bit elliptic curve is slightly better than their results. Please note that the timing figure for our ECC implementation is for a 169-bit elliptic curve with a higher security level, whereas the others’ works use the the smaller ordered 159-bit, 160-bit and 163-bit elliptic curves.

## 5. Conclusions

We implemented ECC on the MSP430 microcontroller, which is a widely used microcontroller in WSNs, by using Edwards curves for point arithmetic and the number theoretic transform for the underlying finite field multiplication and squaring operations. In our work, we realized a novel implementation of the fast Fourier transform over $GF({\left(2^{13}-1\right)}^{13})$ to speed up the number theoretic transform on the MSP430 microcontroller. Furthermore, for the point addition and doubling operations on Edwards curves, we introduced optimized formulae where some arithmetic operations are eliminated by taking advantage of the number theoretic transform. Our ECC implementation resulted in comparable or better timing values than the existing work in the literature on the same microcontroller. Please note that the techniques introduced in this paper can be applied to ECC implementations over other elliptic curves with more efficient formulae for point arithmetic. We identify the application of the introduced techniques to ECC implementations on other elliptic curves, such as Montgomery curves or twisted Edwards curves, as directions for future research.

## Figures and Tables

**Table 1:** Our timings for $GF({\left(2^{13}-1\right)}^{13})$ arithmetic and ECC operations on MSP430F1611 @ 8 MHz.

Operation			Timing
Forward NTT	(Algorithm 4)		0.21 ms
Inverse NTT	(Algorithm 5)		0.44 ms
NTT Squaring			0.78 ms
NTT Multiplication			1.02 ms
ECC Point Doubling	(Algorithm 6)		5.63 ms
ECC Point Addition	(Algorithm 7)		9.64 ms
NAF4 ECC Random Point Multiplication			1.31 s
Comb4 ECC Fixed Point Multiplication			0.65 s

**Table 2:** Timings for ECC random point multiplication.

Microcontroller	Field	Method	Timing
MSP430F1611 @ 8 MHz [67]	$F_{P159}$	Montgomery ladder	0.48 s
MSP430F1611 @ 8 MHz [68]	$F_{P160}$	4NAF	0.58 s
MSP430F1611 @ 8 MHz **(This work)**	$F_{{\left(2^{13}-1\right)}^{13}}$	4NAF	1.31 s
MSP430F149 @ 8 MHz [20]	$F_{{\left(2^{13}-1\right)}^{13}}$	4NAF	1.55 s
MSP430F1611 @ 8 MHz [24]	$F_{P160}$	-	1.60 s
MSP430F1611 @ 8 MHz [69]	$F_{P160}$	-	3.51 s

**Table 3:** Timings for ECC fixed point multiplication.

Microcontroller	Field	Method	Timing
MSP430F1611 @ 8 MHz [67]	$F_{P159}$	Comb	0.24 s
MSP430F1611 @ 8 MHz [68]	$F_{P160}$	4NAF	0.52 s
MSP430F1611 @ 8 MHz **(This work)**	$F_{{\left(2^{13}-1\right)}^{13}}$	Comb	0.65 s
MSP430F1611 @ 8 MHz [22]	$F_{P160}$	Comb	0.72 s
MSP430F1611 @ 8 MHz [22]	$F_{2^{163}}$	Comb	1.04 s
MSP430F1611 @ 8 MHz [70]	$F_{P160}$	-	1.09 s
MSP430F1611 @ 8 MHz [24]	$F_{P160}$	Sliding Window	1.44 s
MSP430F1611 @ 8 MHz [71]	$F_{P160}$	Sliding window	1.58 s
